# The risk of thyroid cancer after hysterectomy and oophorectomy: a meta-analysis

**DOI:** 10.3389/fonc.2024.1446303

**Published:** 2024-09-24

**Authors:** Shenguang Fu, Yiping Lu, Yibo Liu

**Affiliations:** ^1^ Beijing University of Chinese Medicine, Beijing, China; ^2^ Beijing Hospital of Traditional Chinese Medicine, Capital Medical University, Beijing, China

**Keywords:** thyroid cancer, hysterectomy, oophorectomy, meta-analysis, systematic reviews

## Abstract

**Objectives:**

The purpose of this meta-analysis is to assess whether there is an association between hysterectomy and oophorectomy and risk of primary thyroid cancer.

**Methods:**

PubMed, Cochrane Library, Embase, and Web of Science were searched for eligible studies published from database inception to May 13, 2024, using medical subject headings (MeSH) and keywords. All statistical analyses were performed using Stata statistical software (version 14.0). If P > 0.1 and I^2^ ≤ 50%, a fixed-effects model was adopted. If I^2^ > 50% a random-effects model was adopted. The funnel plot and Egger’s test were used to evaluate publication bias.

**Results:**

A total of 11 studies explored the association between a history of hysterectomy, oophorectomy and the risk of thyroid cancer. The pooling analysis shows that a history of hysterectomy, oophorectomy is associated with an increased risk of thyroid cancer (HR = 1.597; 95% CI: 1.467-1.738; I^2^ = 57.1%, P = 0.01 < 0.1). In the subgroup analysis, a follow-up duration exceeding 20 years is linked to an elevated risk of thyroid cancer (HR = 1.772; 95% CI: 1.301-2.414; I² = 81.70%, P = 0.004 > 0.001). Hysterectomy combined with salpingo-oophorectomy is associated with a higher risk of thyroid cancer incidence (HR = 1.633; 95% CI: 1.449-1.841; I² = 51.10%, P = 0.069 > 0.001). Studies that balanced smoking, alcohol consumption, and history of thyroid disease demonstrated an association between hysterectomy and increased risk of thyroid disease (HR = 1.734; 95% CI: 1.591-1.891; I² = 31.30%, P = 0.225 > 0.001).

**Conclusions:**

Our meta-analysis reveals a heightened risk of primary thyroid cancer following hysterectomy and oophorectomy. These findings underscore the importance of considering potential cancer risks when determining surgical approaches and implementing preventive measures prior to these procedures.

The meta-analysis was conducted in adherence to the guidelines outlined in the Preferred Reporting Items for Systematic Reviews and Meta-Analyses (PRISMA) (1). The protocol was pre-registered on the International Prospective Register of Systematic Reviews (PROSPERO) platform, with the registration number CRD42024546451.

**Systematic review registration:**

https://www.crd.york.ac.uk/PROSPERO/#recordDetails, identifier CRD42024546451.

## 1 Introduction

Thyroid cancer arises from malignant tumors originating in the thyroid follicular epithelium or adjacent follicular epithelial cells, making it the most prevalent malignancy in the head and neck region (2). Over the past three decades, there has been a notable uptick in thyroid cancer incidence. In the United States alone, around 43,800 new cases were reported among women in 2022, marking a threefold higher incidence rate compared to men (3). In China, thyroid cancer ranks as the fourth most common cancer among women, with an annual incidence increase of 12.4% up to 2020 (4, 5). This surge in thyroid cancer cases is primarily attributed to overdiagnosis. Known risk factors (6–10) for developing thyroid cancer include a history of radiation exposure, familial thyroid disease, mutations in the RET gene, goiter history, female gender, and Asian ethnicity. Intriguingly, clinical observations suggest that patients who have undergone hysterectomy and oophorectomy exhibit a higher incidence of thyroid cancer compared to those who haven’t undergone these procedures. Despite advancements in treatment modalities, surgical intervention remains the cornerstone in managing thyroid cancer, often complemented by postoperative endocrine therapy and radioactive iodine treatment (11).

In the field of gynecology, hysterectomy and oophorectomy are standard procedures for addressing malignant conditions affecting the female reproductive system (12, 13). The observed changes in postoperative reproductive hormone levels among patients undergoing these surgeries may potentially increase the risk of thyroid cancer in clinical contexts (14, 15). Currently, there is a dearth of literature exploring whether a positive correlation exists between the two factors. Our aim is to fill this gap by conducting a meta-analysis, consolidating pertinent cohort and case-control studies to investigate the potential link between hysterectomy, oophorectomy, and the heightened risk of thyroid cancer.

## 2 Methods

The meta-analysis was conducted in adherence to the guidelines outlined in the Preferred Reporting Items for Systematic Reviews and Meta-Analyses (PRISMA) (1). The protocol was pre-registered on the International Prospective Register of Systematic Reviews (PROSPERO) platform, with the registration number CRD42024546451.

### 2.1 Data sources

We conducted a comprehensive search across four medical databases—PubMed, Cochrane Library, Embase, and Web of Science—to identify cohort studies and case-control studies published from the inception of each database up to May 13, 2024. Our search strategy imposed no language restrictions and employed a combination of medical subject headings and keywords. The search terms encompassed various aspects of thyroid neoplasms, including Thyroid Neoplasm, Thyroid Carcinoma*, Cancer of Thyroid, Thyroid Cancer*, Cancer of the Thyroid, Thyroid Adenoma*, as well as terms related to surgical procedures such as Oophorectom*, Female Castration*, Bilateral Ovariectom*, and Hysterectom*. The detailed search strategy used for PubMed can be found in **Supplementary Table 1**. Additionally, we scrutinized the reference lists of included studies and previously published meta-analyses (16, 17) to identify any further relevant trials.

### 2.2 Eligibility criteria

The selection of trials was based on specific eligibility criteria, including: (1) Cohort studies or case-control studies; (2) Studies examining the correlation between hysterectomy, oophorectomy, and the incidence of thyroid cancer. For the purposes of this meta-analysis, “thyroid cancer” referred to “primary thyroid tumors diagnosed during follow-up.” The outcome measure selected was the overall occurrence of thyroid cancer.

Trials were excluded if they lacked hazard ratio (HR), odds ratio (OR), or relative rate (RR) estimates along with their corresponding 95% confidence intervals (CI). In cases where multiple studies presented data from the same cohort, we prioritized inclusion of the study with the longest follow-up period or the largest participant sample size. Additionally, conference abstracts, study protocols, duplicate publications, and studies lacking relevant outcomes were also excluded from consideration.

### 2.3 Study selection

Two reviewers (SGF and YBL) independently screened the literature according to the eligibility and exclusion criteria. Initially, duplicate and irrelevant articles were excluded based on their titles and abstracts. Subsequently, the full texts of potentially eligible articles were retrieved and carefully reviewed to identify all relevant studies. Any discrepancies or disagreements were resolved through discussion with a third reviewer (YPL), who served as an arbitrator.

### 2.4 Data extraction

Data extraction was conducted independently by the aforementioned reviewers (SGF and YBL), who adhered to established guidelines for systematic reviews and meta-analyses (18). Utilizing pre-designed forms, they extracted relevant information such as the first author, year of publication, study design, sample size, duration of follow-up, age distribution, diagnosis of thyroid cancers, and adjusted confounders. Any discrepancies were resolved through discussion with YPL to achieve consensus.

### 2.5 Risk of bias assessment

The Newcastle-Ottawa Scale (NOS) (19) was employed to evaluate the quality of cohort studies. Scores ranged from 0 to 9 points for cohort or case-control studies, with four points allocated for selection of participants and measurement of exposure, two points for comparability, and three points for assessment of outcomes and adequacy of follow-up. Higher scores denoted a higher quality of study. Scores falling within the ranges of 0–3, 4–6, and 7–9 were interpreted as indicating low, moderate, and high quality, respectively.

### 2.6 Statistical analysis

The adjusted HR and 95% CI from each study were used to assess the association between hysterectomy, oophorectomy and thyroid cancer. The Q-test and the I2-values were used to evaluate heterogeneity. If P > 0.1 and I2 ≤ 50%, a fixed-effects model was adopted. If I2 > 50% (which indicated great heterogeneity), a random-effects model was adopted. The sensitivity analysis was performed by excluding one study each time and rerunning to verify the robustness of the overall effects (20, 21). The funnel plot was visually examined to confirm the presence of publication bias, while Egger’s regression test was employed to statistically evaluate such bias. Subgroup analyses were conducted based on the history of hysterectomy and oophorectomy, duration of follow-up, and whether smoking, alcohol consumption, and a history of thyroid disease were considered confounding factors. All statistical analyses were carried out using Stata statistical software version 14.0 (Stata Corp, College Station, Texas).

## 3 Results

### 3.1 Literature search

**Figure 1:**
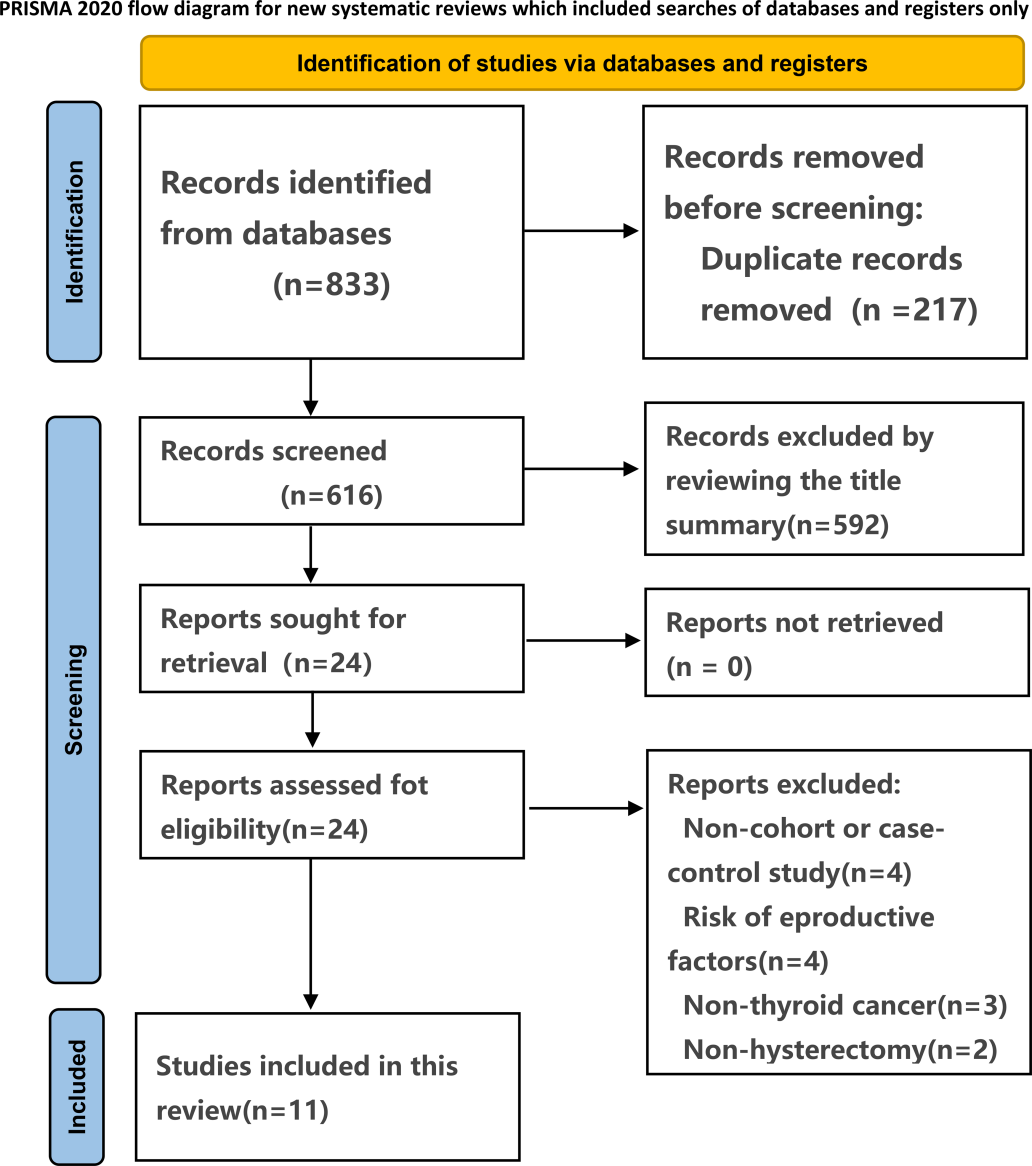
Studies screening process.

The systematic search of observational studies published before May 13, 2024, yielded 833 results. Following the screening of titles and abstracts, 24 articles were deemed potentially relevant. 11 studies (22–32) were included after full text review. The selection process is presented in **Figure 1**.

### 3.2 Study characteristics

This meta-analysis comprised 10 cohort studies and 1 case-control study, spanning from 1997 to 2024. The average follow-up duration varied between 6 and 27 years. Although the adjusted confounders differed slightly across studies, adjusted estimates were accessible for nearly all trials. **Table 1** presents the principal characteristics of the included studies.

**Table 1:** Basic characteristics of the included studies.

Author	Year	Country	Thyroid cancer cases	cases after surgery	Follow-up years	Age(Mean ± SD)	Confounders adjusted	NOS Scores
D. Altman (22)	2016	Sweden	119	111595	11.1 years	50.4(11.1)	age, education, parity, age at surgery	9
A. Guenego (23)	2019	France	412	9143	21.4 years	53.1(6.2)	age (time-scale), smoking status, history of dysthyroid, history of other benign thyroid disease, BMI	8
Y. J. Jin (24)	2021	Korea	1303	13251	12 years	52.2(7.1)	age, body mass index (BMI), hypertension, diabetes mellitus, thyroid disease histories, occupation, smoking, and drinking alcohol, hysterectomy, oophorectomy, the number of children, and the use of oral contraceptive	7
G. C. Kabat (25)	2012	USA	343	NA	12.7 years	NA	Age at menarche, age at menopause, age at first birth, age at last birth, parity, duration of breastfeeding, miscarriages, still-births, hysterectomy, and bilateral oophorectomy	8
M. Kim (26)	2021	Korea	12959	78961	12.7 years	45.9(7.8)	Age, BMI, smoking status, alcohol consumption, indication for surgery, hospital visit, health screening exam, comorbidity, family history of cancer, history of thyroid diseases	8
J. Luo (27)	2016	China	344	46852	14.4 years	63.0(7.2)	Age, race, smoking status, alcohol intake, history of hormone therapy use, family history of cancer, history of thyroid disease, age at hysterectomy	8
R. Luoto (28)	1997	Finland	84	25379	20.5 years	NA	Education, parity and follow-up	7
R. Luoto (29)	2003	Finland	236	93282	6 years	NA	NA	7
S. T. Rahman (30)	2021	Australia	730	285	NA	NA	Age at the reference date, educational attainment, IRSD score, BMI, history of endometriosis, fibroids, PCOS	8
T. S. Tai (31)	2024	Chinese Taiwan	313	29577	10.03 years	47.96(9.67)	Age, urbanization levels, income level, and comorbidities	7
L. F. Wilson (32)	2021	Australia	1095	102785	27 years	NA	Age at entry, parity, remoteness of residence, and IRSD quintile, endometriosis, uterine fibroids, and genital prolapse variables	7

### 3.3 Quality assessment

Based on the NOS criteria, the average score across all included studies was 7.64, with each study scoring 7 or higher. This indicates that all cohort studies included in this meta-analysis were of high quality. The individual scores of the included studies are detailed in **Table 1**.

### 3.4 Hysterectomy, oophorectomy and the risk of thyroid cancer

**Figure 2:**
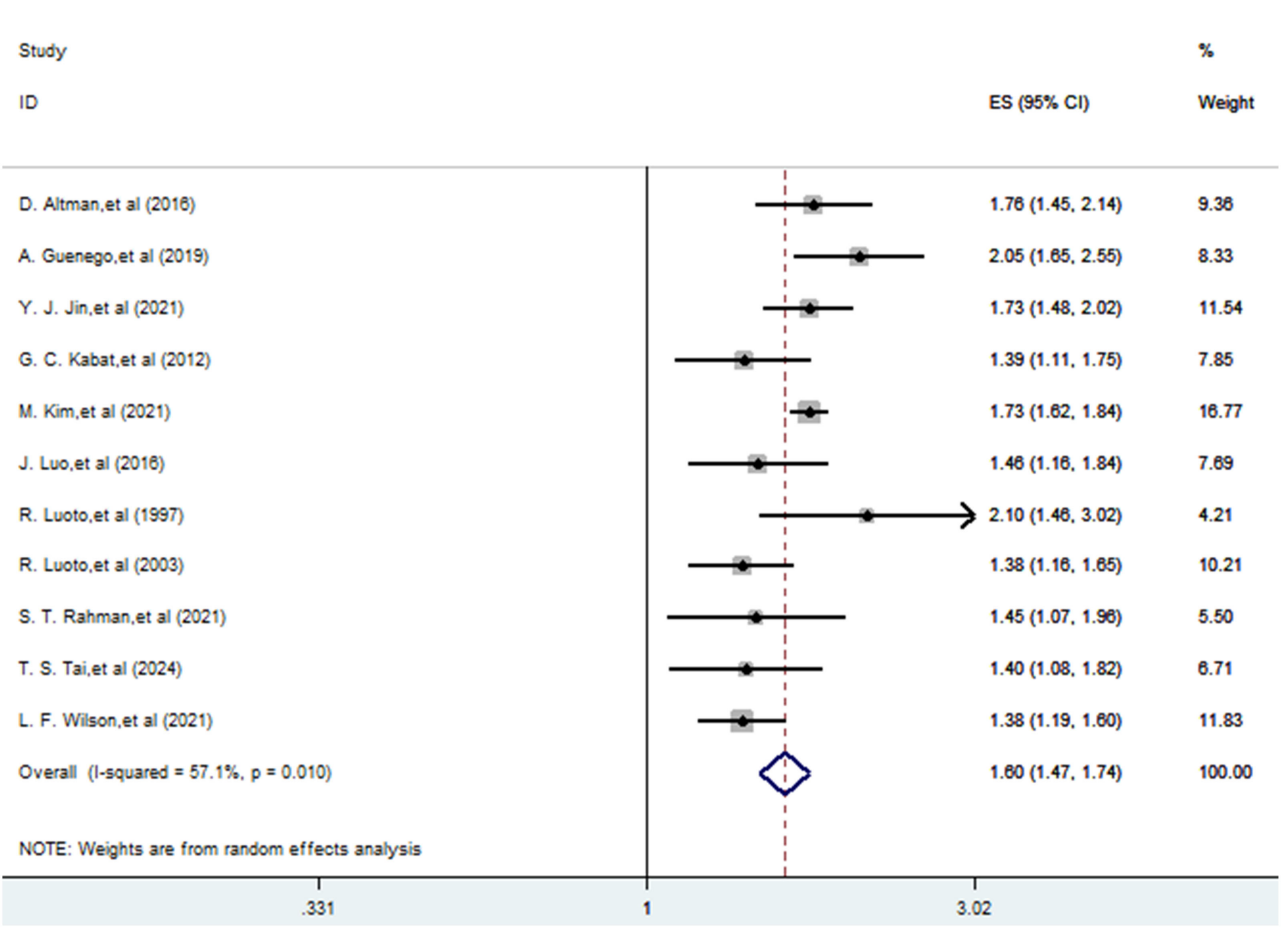
Meta-analysis of the risk of thyroid cancer caused by hysterectomy, oophorectomy.

A total of 11 studies (22–32) explored the association between a history of hysterectomy, oophorectomy and the risk of thyroid cancer. The pooling analysis shows that a history of hysterectomy, oophorectomy is associated with an increased risk of thyroid cancer (HR = 1.597; 95% CI: 1.467-1.738; I2 = 57.1%, P = 0.01; **Figure 2**). Sensitivity analysis showed that none of the individual studies reversed the pooled-effect size, which means that the results are robust (**Supplementary Figure A**).

### 3.5 Subgroup analysis

We performed a subgroup analysis based on surgery type, follow-up duration, and adjusted confounders (**Table 2**). Notably, a follow-up duration exceeding 20 years is linked to an elevated risk of thyroid cancer (HR = 1.772; 95% CI: 1.301-2.414; I² = 81.70%). Hysterectomy combined with salpingo-oophorectomy is associated with a higher risk of thyroid cancer incidence (HR = 1.633; 95% CI: 1.449-1.841; I² = 51.10%). Studies that balanced smoking, alcohol consumption, and history of thyroid disease demonstrated an association between hysterectomy and increased risk of thyroid disease incidence (HR = 1.734; 95% CI: 1.591-1.891; I² = 31.30%).

**Table 2:** Subgroup analysis for risk of thyroid cancer caused by hysterectomy, oophorectomy.

Subgroups	Included Studies	HR (95% CI)	Heterogeneity	
			I²	P-values
With BSO or not				
HT^a^ with BSO^b^	6	1.633(1.449-1.841)	51.10%	0.069
HT only	5	1.554(1.344-1.795)	69.40%	0.011
Follow-up Years				
<10	1	1.380(1.156-1.648)	0.00%	0.000
≥10, <15	6	1.642(1.518-1.777)	29.10%	0.216
≥20	3	1.772(1.301-2.414)	81.70%	0.004
Confounders adjusted^c^				
Adjusted	4	1.734(1.591-1.891)	31.30%	0.225
No Adjusted	7	1.489(1.348-1.644)	29.80%	0.201

a, hysterectomy; b, bilateral salpingo-oophorectomy; c, whether smoking status, alcohol consumption, and history of thyroid disease were balanced.

### 3.6 Publication bias

**Figure 3:**
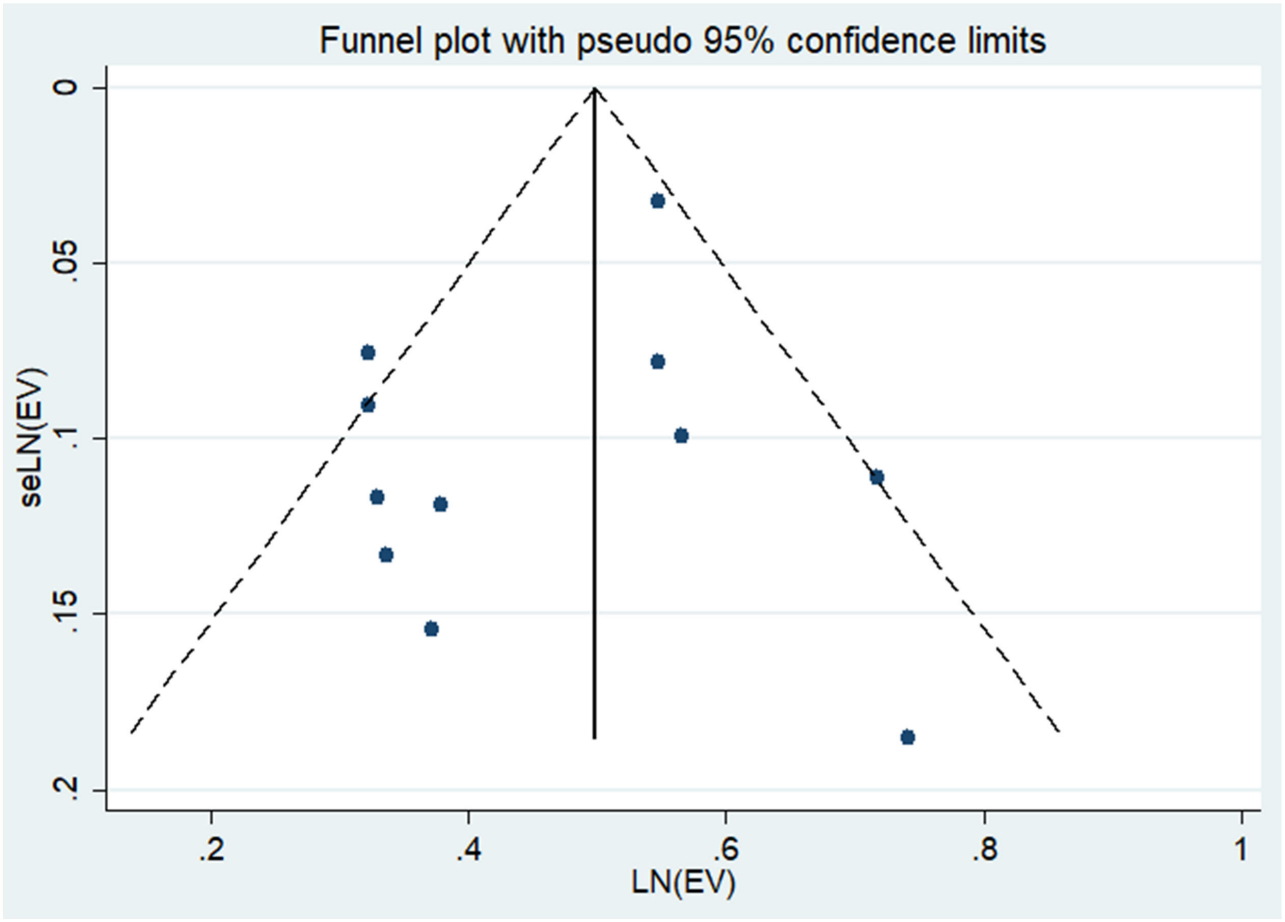
Publication bias of the risk of thyroid cancer caused by hysterectomy, Oophorectomy.

Upon visual examination of the funnel plot, there was no discernible indication of significant publication bias regarding the outcome of Hysterectomy, Oophorectomy, and the Risk of Thyroid Cancer (see **Figure 3**). Additionally, Egger’s regression test (P = 0.34 > 0.05) provided further evidence suggesting the absence of publication bias in our meta-analysis.

## 4 Discussion

This meta-analysis comprised 10 cohort studies and 1 case-control studies (22–32), providing a comprehensive assessment of the association between hysterectomy, oophorectomy, and thyroid cancer incidence. We found that individuals undergoing these surgeries had a significantly increased risk of primary thyroid cancer compared to those without surgery, with a 1.597-fold increase. This suggests that hysterectomy and oophorectomy may serve as independent risk factors for thyroid cancer.

A previous meta-analysis (17) investigated the relationship between hysterectomy and thyroid cancer. The results indicated that hysterectomy increases the risk of thyroid cancer incidence. Additionally, it did not find any other confounding factors associated with thyroid cancer incidence rates. In contrast, in the current analysis, we included more recent studies and conducted subgroup analyses based on whether oophorectomy was included, follow-up duration, and whether smoking, alcohol consumption, and history of thyroid disease were balanced, to provide robust evidence for the association between hysterectomy, oophorectomy, and thyroid cancer.

Although the precise reasons for this association remain uncertain, one potential explanation is that the uterus, beyond its reproductive function, also produces factors that influence cancer pathways. Consequently, hysterectomy might directly impact the thyroid cancer pathway by disrupting the synthesis and release of substances affecting the thyroid, such as growth factors, P substance, and vasoactive peptides (33, 34). Moreover, hysterectomy is frequently used to manage benign gynecological conditions linked to elevated estrogen levels, which themselves could be associated with thyroid cancer. Thus, the heightened risk of thyroid cancer post-hysterectomy could be attributed to either the surgical procedure itself or its potential correlation with the conditions necessitating surgery. Gynecological surgery can lead to changes in lipid peroxidation levels, which may induce DNA damage and promote mutations in oncogenes and tumor suppressor genes (35). One interesting finding (36) indicate that the ovaries are a significant source of androgens throughout a woman’s entire lifespan. Since both hysterectomy and oophorectomy are likely to cause significant changes in hormone levels, they also influence the risk of hormone-related cancers. Nevertheless, further research is imperative to comprehensively grasp this relationship (25, 29, 33). Additionally, a piece of literature suggests that mutations in the BRAF V600E or RAS genes lead to constitutive activation of the MAPK signaling pathway in thyroid follicular cells, driving the origin of thyroid tumors (37).

Our meta-analysis synthesized existing evidence, affirming the relationship between hysterectomy, oophorectomy, and the risk of thyroid cancer. This suggests a need for heightened awareness of thyroid cancer risk among postoperative patients, facilitating early identification of high-risk individuals for thyroid cancer. However, this study also has certain limitations. Most of the studies we included were cohort studies and there was only one case-control study, which may inevitably present problems of confounding bias and recall bias in observational studies. There was a degree of heterogeneity in the results of the meta-analyses, so sensitivity analyses and bias tests were conducted to ensure the reliability of the findings. In addition, there are very few mechanistic studies exploring the risk of thyroid cancer after hysterectomy and oophorectomy, and further work is needed to analyze this association at the mechanistic level.

## 5 Conclusions

This meta-analysis suggest that the combination of hysterectomy and oophorectomy raises the likelihood of developing primary thyroid cancer. Nevertheless, delving deeper into the underlying mechanisms of this correlation, particularly from a pathological and physiological perspective, is imperative.

## 6 Limitation analysis

Since the included studies did not provide detailed stratification of the age at which patients underwent hysterectomy or oophorectomy, we were unable to perform a subgroup analysis based on surgical age. The question of whether the age at which patients undergo hysterectomy or oophorectomy is associated with an increased risk of thyroid cancer remains to be explored further.

## Data Availability

The original contributions presented in the study are included in the article/**Supplementary Material**. Further inquiries can be directed to the corresponding author/s.
