# Supplementary material for: The risk of thyroid cancer after hysterectomy and oophorectomy: a meta-analysis
Source: Front Oncol. 2024 Sep 24;14:1446303. doi: 10.3389/fonc.2024.1446303 (PMC11460577; doi:10.3389/fonc.2024.1446303)
Supplement: Supplementary file 1 [file DataSheet1.pdf]

Table 1: PubMed

No.	Content	Result
#1	Search: **"Thyroid Neoplasms"[Mesh]** Sort by: **Most Recent**	63495
#2	Search: **(((((Thyroid Neoplasm[Title/Abstract]) OR (Thyroid Carcinoma*[Title/Abstract])) OR (Cancer of Thyroid[Title/Abstract])) OR (Thyroid Cancer*[Title/Abstract])) OR (Cancer of the Thyroid[Title/Abstract])) OR (Thyroid Adenoma*[Title/Abstract])** Sort by: **Most Recent**	53773
#3	Search: **("Thyroid Neoplasms"[Mesh]) OR ((((((Thyroid Neoplasm[Title/Abstract]) OR (Thyroid Carcinoma*[Title/Abstract])) OR (Cancer of Thyroid[Title/Abstract])) OR (Thyroid Cancer*[Title/Abstract])) OR (Cancer of the Thyroid[Title/Abstract])) OR (Thyroid Adenoma*[Title/Abstract]))** Sort by: **Most Recent**	78197
#4	Search: **"Ovariectomy"[Mesh]** Sort by: **Most Recent**	27630
#5	Search: **((Oophorectom*[Title/Abstract]) OR (Female Castration*[Title/Abstract])) OR (Bilateral Ovariectom*[Title/Abstract])** Sort by: **Most Recent**	12682
#6	Search: **("Ovariectomy"[Mesh]) OR (((Oophorectom*[Title/Abstract]) OR (Female Castration*[Title/Abstract])) OR (Bilateral Ovariectom*[Title/Abstract]))** Sort by: **Most Recent**	35736
#7	Search: **"Hysterectomy"[Mesh]** Sort by: **Most Recent**	34339
#8	Search: **Hysterectom*[Title/Abstract]** Sort by: **Most Recent**	44559
#9	Search: **("Hysterectomy"[Mesh]) OR (Hysterectom*[Title/Abstract])** Sort by: **Most Recent**	56198
#10	Search: **(("Ovariectomy"[Mesh]) OR (((Oophorectom*[Title/Abstract]) OR (Female Castration*[Title/Abstract])) OR (Bilateral**	84031

	**Ovariectom*[Title/Abstract]))) OR (("Hysterectomy"[Mesh]) OR (Hysterectom*[Title/Abstract]))** Sort by: **Most Recent**	
#11	Search: **(("Thyroid Neoplasms"[Mesh]) OR ((((((Thyroid Neoplasm[Title/Abstract]) OR (Thyroid Carcinoma*[Title/Abstract])) OR (Cancer of Thyroid[Title/Abstract])) OR (Thyroid Cancer*[Title/Abstract])) OR (Cancer of the Thyroid[Title/Abstract])) OR (Thyroid Adenoma*[Title/Abstract]))) AND ((("Ovariectomy"[Mesh]) OR (((Oophorectom*[Title/Abstract]) OR (Female Castration*[Title/Abstract])) OR (Bilateral Ovariectom*[Title/Abstract]))) OR (("Hysterectomy"[Mesh]) OR (Hysterectom*[Title/Abstract])))** Sort by: **Most Recent**	149

Table 2 Embase

No.	Content	Result
#1	'thyroid tumor'**/exp**	115769
#2	'thyroid neoplasm'**:ab,ti OR** 'thyroid carcinoma*'**:ab,ti OR** 'cancer of thyroid'**:ab,ti OR** 'thyroid cancer*'**:ab,ti OR** 'cancer of the thyroid'**:ab,ti OR** 'thyroid adenoma*'**:ab,ti**	72877
#3	**#1 OR #2**	121187
#4	'ovariectomy'**/exp**	43393
#5	oophorectom***:ab,ti OR** 'female castration*'**:ab,ti OR** 'bilateral ovariectom*'**:ab,ti**	19062
#6	**#4 OR #5**	54311
#7	'hysterectomy'**/exp**	96433
#8	hysterectom***:ab,ti**	70729
#9	**#7 OR #8**	107772
#10	**#6 OR #9**	149006
#11	**#3 AND #10**	526

Table 3 Cochran Library

No.	Content	Result
#1	**MeSH descriptor: [Thyroid Neoplasms] explode all trees**	1002
#2	**(Thyroid Neoplasm):ti,ab,kw OR (Thyroid Carcinoma*):ti,ab,kw OR (Cancer of Thyroid):ti,ab,kw OR (Thyroid Cancer*):ti,ab,kw OR (Cancer of the Thyroid):ti,ab,kw**	2535
#3	**(Thyroid Adenoma*):ti,ab,kw**	141
#4	**#1 OR #2 OR #3**	2856
#5	**MeSH descriptor: [Ovariectomy] explode all trees**	418
#6	**(Oophorectom*):ti,ab,kw OR (Female Castration*):ti,ab,kw OR (Bilateral Ovariectom*):ti,ab,kw**	2320
#7	**#5 OR #6**	2500
#8	**MeSH descriptor: [Hysterectomy] explode all trees**	2482
#9	**(Hysterectom*):ti,ab,kw**	9107
#10	**#8 OR #9**	9109
#11	**#7 OR #10**	10297
#12	**#4 AND #11**	19

Table 4 Web of science

No.	Content	Result
#1	**Thyroid Neoplasm* (Topic) or Thyroid Carcinoma* (Topic) or Cancer of Thyroid (Topic) or Thyroid Cancer* (Topic) or Cancer of the Thyroid (Topic) or Thyroid Adenoma* (Topic)**	60617
#2	**Ovariectomy (Topic) or Oophorectom* (Topic) or Female Castration* (Topic) or Bilateral Ovariectom* (Topic)**	17303
#3	**Hysterectom* (Topic)**	28775
#4	**#2 OR #3**	42829
	**#1 AND #4**	139
