# Supplementary figures and images for: The risk of thyroid cancer after hysterectomy and oophorectomy: a meta-analysis
Source: Front Oncol. 2024 Sep 24;14:1446303. doi: 10.3389/fonc.2024.1446303 (PMC11460577; doi:10.3389/fonc.2024.1446303)

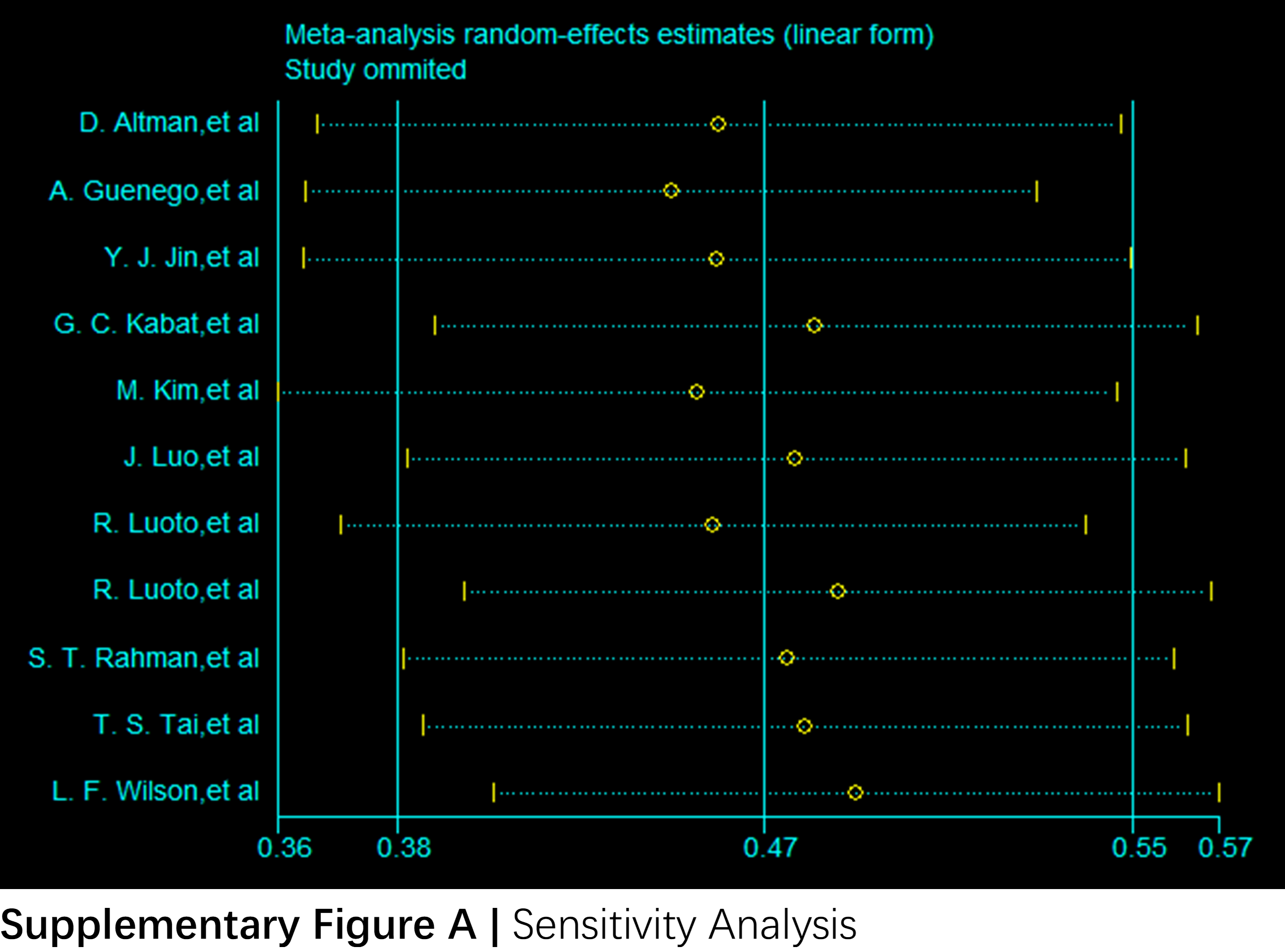

Supplement: Supplementary file 2 [file Image1.tif]
